# ECOG-ACRIN 2399: analysis of patient related outcomes after Chemoradiation for locally advanced head and neck Cancer

**DOI:** 10.1186/s41199-020-00059-1

**Published:** 2020-12-22

**Authors:** Anthony Cmelak, Mary S. Dietrich, Shuli Li, Sheila Ridner, Arlene Forastiere, Barbara A. Burtness, David Cella, Barbara A. Murphy

**Affiliations:** 1 Department of Radiation Oncology, Vanderbilt University, B-1003 Preston Research Building, 2220 Pierce Avenue, Nashville, TN 37232-5671 USA; 2 Dana Farber Cancer Institute, Boston, MA USA; 3 Johns Hopkins University, Baltimore, MD USA; 4 Yale University, New Haven, CT USA; 5 Northwestern University, Evanston, IL USA

**Keywords:** Squamous cell cancer, Head and neck, Induction chemotherapy, Organ preservation, VHNSS, PRO

## Abstract

**Background:**

We conducted a correlative study for E2399, a function preservation trial for resectable locally advanced oropharynx and larynx cancer, to prospectively assess effects of chemoradiation (CCR) on quality of life (QOL), swallowing and voice. We correlated the results of swallow assessments done via questionnaires and objective assessments by modified barium swallow (MBS).

**Methods:**

The Functional Assessment of Cancer-HN (FACT-HN), the Performance Status Scale – Head and Neck (PSS-HN), swallow assessments (including modified barium swallow studies), and voice assessments: Voice Handicap Index (VHI), the Voice Disability Assessment (VDA), and American Speech-Language Hearing Association’s Functional Communication Measure (FCM) were conducted at baseline and periodically post-treatment for 2 years.

**Results:**

Baseline QOL and swallowing function predicted overall survival. Patients experienced a marked decrease in QOL, swallowing, and speech post CCR although the decrease in vocal function was modest. Function and QOL returned towards baseline in the majority of patients by 12 months post treatment. Less than 10% of patients had severe dysphagia and were PEG dependent at 12 months post treatment. There was a high degree of correlation between the FACT-HN and PSS-HN swallow items. Statistically significant correlations were found between subjective and objective measures of swallow function.

**Conclusions:**

Patients experience marked loss in swallowing function post CCR which returned to baseline in the majority of patients. The correlations between the FCM and self-report swallow items on the PSS and FACT-HN appear to be sufficiently strong to justify their use as a surrogate marker for swallowing disability in large therapeutic trials.

## Introduction

An increasing number of patients with resectable cancer of the oropharynx or larynx are undergoing concomitant chemotherapy and radiation (CCR) in an effort to maximize survival and preserve function during and subsequent to cancer treatment [1]. CCR results in improved oncologic treatment outcomes relative to radiation alone; however, effects on swallowing and speech function are incompletely characterized and may adversely impact long-term quality of life (QOL) [2, 3]. Swallow and voice function can be assessed both by patient report and by objective measures. The benefits of questionnaires include low cost, ease of administration, and the ability to repeat measures on a frequent basis. Several symptom questionnaires, including the FACT-HN subscale and the EORTC H&N 35 [4, 5] have questions or subscales which address speech and swallowing function. These self-report measures had not been validated against objective measures of function. While objective measures provide a rich source of data, they are costly and require specialized staff. Validation of patient-report instruments that can be used as surrogates for objective measures in large treatment trials is needed.

ECOG-ACRIN undertook the function preservation trial E2399 to evaluate a non-cisplatin induction and sensitization schedule as a potentially less toxic CCR regimen. Patients with locally advanced squamous cell carcinomas of either the oropharynx (OP) or larynx (L) were eligible, and received induction chemotherapy followed by 70Gy CCR and low dose weekly paclitaxel 30 mg/m2. The primary endpoint of the study was organ preservation. Study details and the primary outcome results have been previously published [6]. Within the context of E2399, we conducted correlative studies to assess QOL and functional outcomes, as the underlying purpose of organ preservation is maintenance of QOL and function. The objectives of the QOL assessments were to assess the trajectory of QOL over time and determine whether baseline QOL predicts response and survival. The objectives of the swallowing assessment were multifold: 1) to conduct a longitudinal assessment of swallow function in patients with locally advanced head and neck cancer undergoing CCR as primary treatment, 2) to determine the correlation between two self-report measures of swallow function (Functional Assessment of Cancer Therapy – Head and Neck (FACT-HN) and the Performance Status Scale-Head and Neck (PSS-HN)), 3) to determine the correlation between two measures of swallowing function that incorporate data obtained from a modified barium swallow (MBS) - the Functional Communication Measures and the Dysphagia Outcome Severity Scale (DOSS), and 4) to determine the correlation between the self-report and objective measures of swallow function. The objective of the voice assessment was to conduct a longitudinal assessment of voice function/serviceability by treating physician, and voice quality as assessed by patients undergoing concurrent chemoradiation. The results of these analyses are reported herein.

## Materials and methods

### Study design

Eligibility criteria for E2399, a phase II function preservation trial, included patients with previously untreated resectable, biopsy proven squamous cell carcinoma or the OP and L, stage T2N + M0 or T3-T4N0-3 M0, age ≥ 18, ECOG PS of 0–2, with good organ system function. Patients received induction chemotherapy of paclitaxel 175 mg/m^2^ IV over 3 h followed by carboplatin AUC 6 IV over 30 to 60 min for 2 cycles 21 days apart. At the completion of induction therapy, patients with a tumor response or stable disease went on to receive radiation therapy 70Gy in 35 fractions over 7 weeks with concurrent weekly paclitaxel 30 mg/m^2^ IV. The protocol was approved by the Institutional Review Boards at all Cooperative Group sites. Written informed consent was obtained from all participants. Treatment results and biologic correlative data have previously been published [6, 7].

### QOL and functional assessment

#### Questionnaires

##### Functional assessment Cancer therapy-head neck (FACT-HN)

The FACT-HN consists of 27 items in the FACT-G (version 4) and an 11-item HN module that measures concerns/symptoms associated with head and neck cancer [8–10]. A subscale score is computed by summing across all items, with higher scores reflecting better QOL. The FACT-HN has been used in HNC trials [9] and has demonstrated sensitivity to change in disease status. Validity has been supported in numerous studies [10, 11]. Internal consistency of the subscale is adequate [12]. The Trial Outcome Index (TOI) is a composite score which includes physical, functional, and a HN cancer specific subscale of the FACT. The questionnaire was completed by the patient at baseline, immediately following induction, and at 3, 6, 12, and 24 months post-chemoradiation.

##### Performance status scale head and neck Cancer patients (PSS-HN)

This 3-domain scale evaluates normalcy of diet, eating in public, and speech and has been used in numerous studies of head and neck cancer patients [13, 14]. Validity and reliability has been previously documented [15]. The PSS-HN was administered by the study staff at the same time points as the FACT-HN.

##### Voice handicap index (VHI)

This 30 item patient-reported instrument evaluates function, emotion, and physical areas that may be affected by voice disorders such as those disorders caused by CCR therapy for head and neck cancer [16]. A Likert-like response format is used with 7 possible responses reflecting degree of impairment. Items are summed to provide a total score. Internal consistency of the 30-item scale (r = 0.95) is adequate. The VHI was completed at time points similar to the FACT-HN.

##### Voice desirability assessment (VDA)

Physicians rated ability to communicate, not aesthetic quality of voice, on a linear scale from zero (aphonic) to fifty (perfect voice) [17]. The VDA was also completed at baseline, post-induction, and at 3, 6, 12, and 24 months post treatment.

##### Swallowing assessment

Patients were scheduled for swallow assessment by a Speech and Language Pathologist (SLP) and a MBS and at baseline, 3, 12 and 24 months post treatment. The procedure for conducting the MBS was delineated in the protocol in order to ensure constancy. Prior to enrolling patients on the study each site was required to identify a Speech and Language Pathologist (SLP) who would conduct study related swallow assessment and the MBS studies. The designated SLP was required to review the protocol and pass a brief quiz aimed at ensuring familiarity with the guidelines. The SLP who conducted the assessment and the MBS scored the patients swallowing function using the American Speech-Language Hearing Association’s Functional Communication Measure (FCM), a Medicare standard for scoring swallowing function. The FCM is a seven-point rating scale designed to describe the change in an individual’s swallowing ability over time. The FCM reports global functioning taking into account subjective issues including: diet level, need for compensatory strategies or cueing, and alternate mode of feeding. In addition, videos of the MBS were scored centrally by the study SLP using the Dysphagia Outcome Severity Scale (DOSS). The DOSS uses only data obtained directly from the MBS and concentrates on the identification of physiologic and anatomic abnormalities.

### Statistical considerations

Analyses were conducted using SPSS (Version 26). Descriptive and graphical methods were used to summarize the data distributions. Frequency distributions were used for nominal and ordinal variables, with the exception of age (mean, SD), all continuous data distributions were heavily skewed. Medians and 25th–75th inter-quartile (IQR) were used to summarize those data. Comparisons of baseline patient demographic and clinical characteristics were conducted using the likelihood Chi-Square statistic (nominal and ordinal data) and Mann-Whitney tests (skewed continuous data). Longitudinal tests of change were conducted using generalized linear modeling that adjusted the standard errors for the lack of independence of measurement. This approach also allowed for missing assessments; however, because missing 12-month data was not assumed to be random (i.e., likely survival related) for inclusion in the longitudinal analysis, a patient must have completed both the baseline and the 12-month assessment. Measures of concordance between the two ordinal FCM and DOSS measures were conducted using the symmetric Somer’s *d* statistic. Bivariate associations were assessed using Spearman’s Rho. Maintaining a maximum alpha of .05 was used for making determinations of statistical significance.

## Results

### Patient characteristics

One hundred eleven patients with squamous cell carcinoma of the OP or L were accrued from March 16, 2001 until May 11, 2004. Five patients either refused or were lost to follow-up and one patient did not complete the baseline self-report assessments. Thus 105 (67 OP, 38 L) are included in the analyses. Seventy-one patients completed the baseline and 12 month assessments. Statistically significant differences between the patients with and without 12-month assessments included primary site, T-stage, requirement for salvage surgery, best overall response, performance status, and time to survival (*p* < 0.05, see Table 1).

**Table 1:** Demographic and clinical characteristics of the patients with and without 12-month assessments

Characteristic	Total (*N* = 105) N (%)	12-Month (*N* = 71) N (%)	No 12-Month (*N* = 34) N (%)	*p*-value
Male	86 (81.9)	57 (80.3)	29 (85.3)	0.526
White	88 (83.8)	63 (88.7)	25 (73.5)	0.054
Primary Site				
Larynx	38 (36.2)	20 (28.2)	18 (52.9)	0.014
Pharynx	67 (63.8)	51 (71.8)	16 (47.1)	
T-Stage				
I-II	43 (41.0)	34 (47.9)	9 (26.5)	0.034
III-IV	62 (59.0)	37 (52.1)	25 (73.5)	
N-Stage				
0–1	45 (42.9)	30 (42.3)	15 (44.1)	0.857
2–3	60 (57.1)	41 (57.7)	19 (55.9)	
Surgery dissection (primary site)	14 (13.3)	5 (7.0)	9 (26.5)	0.008
Cell differentiation^a^				
Poorly	35 (38.5)	28 (43.8)	7 (25.9)	0.179
Moderately	48 (52.7)	32 (50.0)	16 (59.3)	
Well	8 (8.8)	4 (6.3)	4 (14.8)	
Best overall response^b^				
Progressive disease	4 (4.3)	2 (2.9)	2 (7.7)	< 0.001
Stable disease	22 (23.4)	7 (10.3)	15 (57.7)	
Partial response	32 (34.0)	27 (39.7)	5 (19.2)	
Complete response	36 (38.3)	32 (47.1)	4 (15.4)	
Weight loss^c^ (previous 6 months)				
> 10% body weight	12 (12.2)	6 (9.2)	6 (18.2)	0.288
5–10% body weight	18 (18.4)	14 (21.5)	4 (12.1)	
< 5% body weight	68 (69.4)	45 (69.2)	23 (69.7)	
Performance Status				
Fully active	50 (47.6)	40 (80.0)	10 (20.0)	0.035
Strenuous restricted	48 (45.7)	27 (56.3)	21 (43.8)	
All self-care	7 (6.7)	4 (57.1)	3 (42.9)	
Time to survival (months)				
> = 24	63 (61.8)	52 (75.4)	11 (33.3)	< 0.001
12–23	26 (25.5)	17 (24.6)	9 (27.3)	
3–11	11 (10.8)	0 (0.0)	11 (33.3)	
< 3	2 (2.0)	0 (0.0)	2 (6.1)	
	Mean (SD)	Mean (SD)	Mean (SD)	
Age	58.9 (10.7)	57.9 (9.7)	60.9 (12.5)	0.198

^a^ *N* = 91; ^b^ *N* = 94; ^c^ *N* = 98

#### Baseline factors associated with survival

Patient, disease characteristics, and treatment outcomes of patient who survived to 12 months and those who died of all causes before the 12 months assessment time point are summarized in Table 2. Statistically significant differences included N-stage, required salvage surgery, best overall response, weight loss in the prior six months before study entry, and age (*p* < 0.05, see Table 2).

**Table 2:** Demographic and clinical characteristics of the patients all patients

Characteristic	Total (N = 105) N (%)	Alive (*N* = 78) N (%)	Deceased (*N* = 27) N (%)	*p*-value
Male	86 (81.9)	63 (80.8)	23 (85.2)	0.601
White	88 (83.8)	66 (84.6)	22 (81.5)	0.706
Primary Site				0.052
Larynx	38 (36.2)	24 (30.8)	14 (51.9)	
Pharynx	67 (63.8)	54 (69.2)	13 (48.1)	
T-Stage				0.630
I-II	43 (41.0)	33 (42.3)	10 (37.0)	
III-IV	62 (59.0)	45 (57.7)	17 (63.0)	
N-Stage				0.035
0–1	45 (42.9)	38 (48.7)	7 (25.9)	
2–3	60 (57.1)	40 (51.3)	20 (74.1)	
Surgery dissection (primary site)	14 (13.3)	7 (9.0)	7 (25.9)	0.035
Cell differentiation^a^				0.486
Poorly	35 (38.5)	26 (39.4)	9 (36.0)	
Moderately	48 (52.7)	33 (50.0)	15 (60.0)	
Well	8 (8.8)	7 (10.6)	1 (4.0)	
Best overall response^b^				< 0.001
Progressive disease	4 (4.3)	1 (1.4)	3 (13.6)	
Stable disease	22 (23.4)	12 (16.7)	10 (45.5)	
Partial response	32 (34.0)	25 (34.7)	7 (31.8)	
Complete response	36 (38.3)	34 (47.2)	2 (9.1)	
Weight loss^c^ (previous 6 months)				0.036
> 10% body weight	12 (12.2)	5 (6.9)	7 (26.9)	
5–10% body weight	18 (18.4)	15 (20.8)	3 (11.5)	
< 5% body weight	68 (69.4)	52 (72.2)	16 (61.5)	
	Mean (SD)	Mean (SD)	Mean (SD)	
Age	58.9 (10.7)	57.5 (9.8)	62.6 (12.4)	0.034

^a^ *N* = 91; ^b^ *N* = 94; ^c^ *N* = 98

### Quality of life

#### Associations of baseline QOL with survival

Summaries of the scores on the QOL measures at entry into the study for patients who survived to 12 months and those who did not are presented in Table 3. Statistically significantly higher scores for those who survived were observed on the FACT physical subscale, as well as the FACT-HN and TOI (*p* < 0.05, see Table 3).

**Table 3:** Baseline QOL measure scores of the patients who survived and those who did not

QOL Measure	Total (*N* = 105) Median (IQR)	Alive (*N* = 78) Median (IQR)	Deceased (*N* = 27) Median (IQR)	*p*-value
FACT				
Physical	24.5 (21,27)	25.0 (22,27)	23.3 (19,25)	0.040
Social	25.0 (21,28)	25.3 (21,28)	24.0 (22,28)	0.624
Emotional	16.0 (13,17)	15.8 (13,17)	16.0 (12,19)	0.377
Functional	19.0 (14,24)	19.3 (16,25)	18.0 (13,20)	0.070
Total	83.1 (71,90)	85.0 (72,93)	80.0 (68,89)	0.116
FACT-HN	27.0 (22,31)	28.0 (24,32)	21.0 (12,28)	< 0.001
TOI	71.0 (58,80)	73.0 (61,81)	62.4 (47,73)	0.002

#### Trajectory of QOL over time

Summaries of the QOL measures (FACT, FACT-HN, TOI) at each time of assessment for the patients with 12-month assessments are displayed in Table 4. Statistically significant changes in QOL over time were found for the emotional subscale of the FACT, the FACT-HN and TOI (*p* < 0.001). Post-hoc analyses indicated that the FACT emotional subscale scores increased from baseline to post induction (*p* < 0.05) and remained steady thereafter. Reductions in FACT-HN scores were found between post-induction and three months (*p* < 0.05). Scores returned towards baseline at 12-months but were still lower than at entry into the study (*p* < 0.05). The TOI scores also demonstrated a decrease between post-induction and three months (*p* < 0.05) with a subsequent return to baseline levels at 12 months.

**Table 4:**
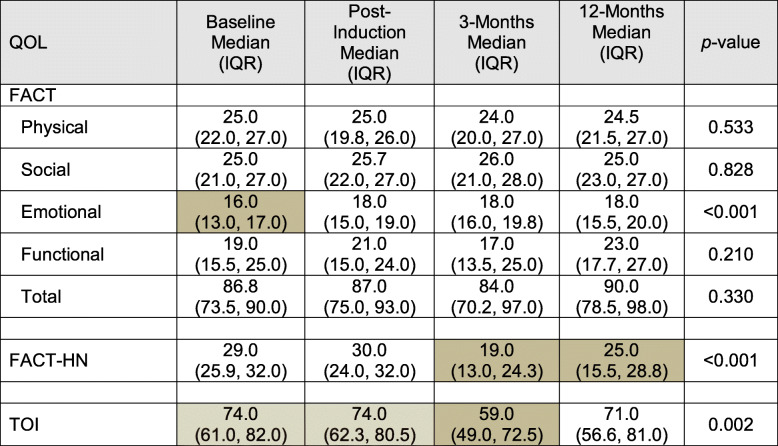
QOL longitudinal summaries over time

Note: Shaded boxes indicate statistically significant pairwise differences in post-hoc analyses

### Swallowing

#### Longitudinal assessment of swallow function

All self-report and objective measures of swallowing demonstrated statically significant changes in function from baseline to 12 months post treatment (*p* < 0.001, Table 5). Post hoc analysis revealed that most of the reduction occurred between baseline and 3 months post treatment. In general, swallow function returned towards baseline by 12 months post treatment, however still remained below baseline levels (*p* < 0.05).

**Table 5:**
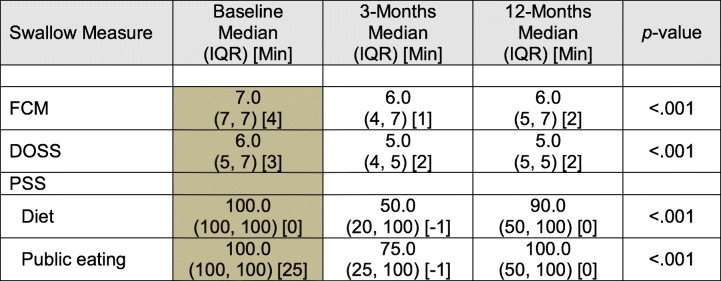
Swallowing longitudinal summaries over time

Note: Shaded boxes indicate statistically significant pairwise differences in post-hoc analyses

#### Concordance of two objective measures of swallow function

**Fig. 1:**
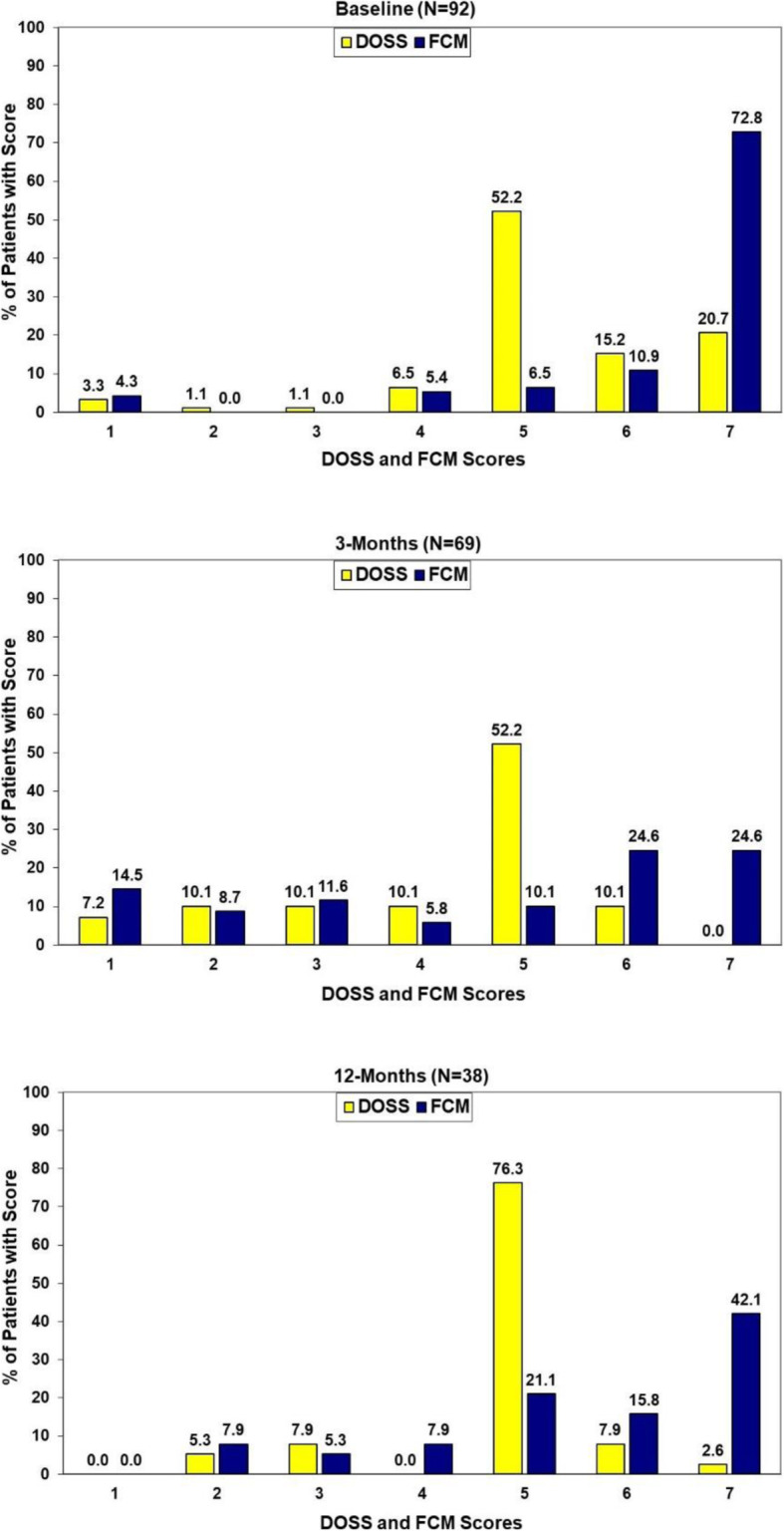
Concordance of DOSS and FCM Assessments at Baseline

Concordance of the two objective measures of swallow function at baseline, 3 and 12 months post treatment are presented in Fig. 1. While statistically significant correlations existed between the two measures at each of these assessment points (*p* < 0.001, *Somer’s d:* baseline = 0.41, 3-months = 0.71, 12-months = 0.53) there clearly were differences in the specific patterns of the scores (baseline: *Χ*^2^_(df = 24)_ = 60.25 *p* < 0.001, 3-months:: *Χ*^2^_(df = 30)_ = 102.62 *p* < 0.001, 12-months: *Χ*^2^_(df = 20)_ = 36.71 *p* = 0.013). (Fig. 1).

#### Correlations among self-report measures of swallow function

**Fig. 2:**
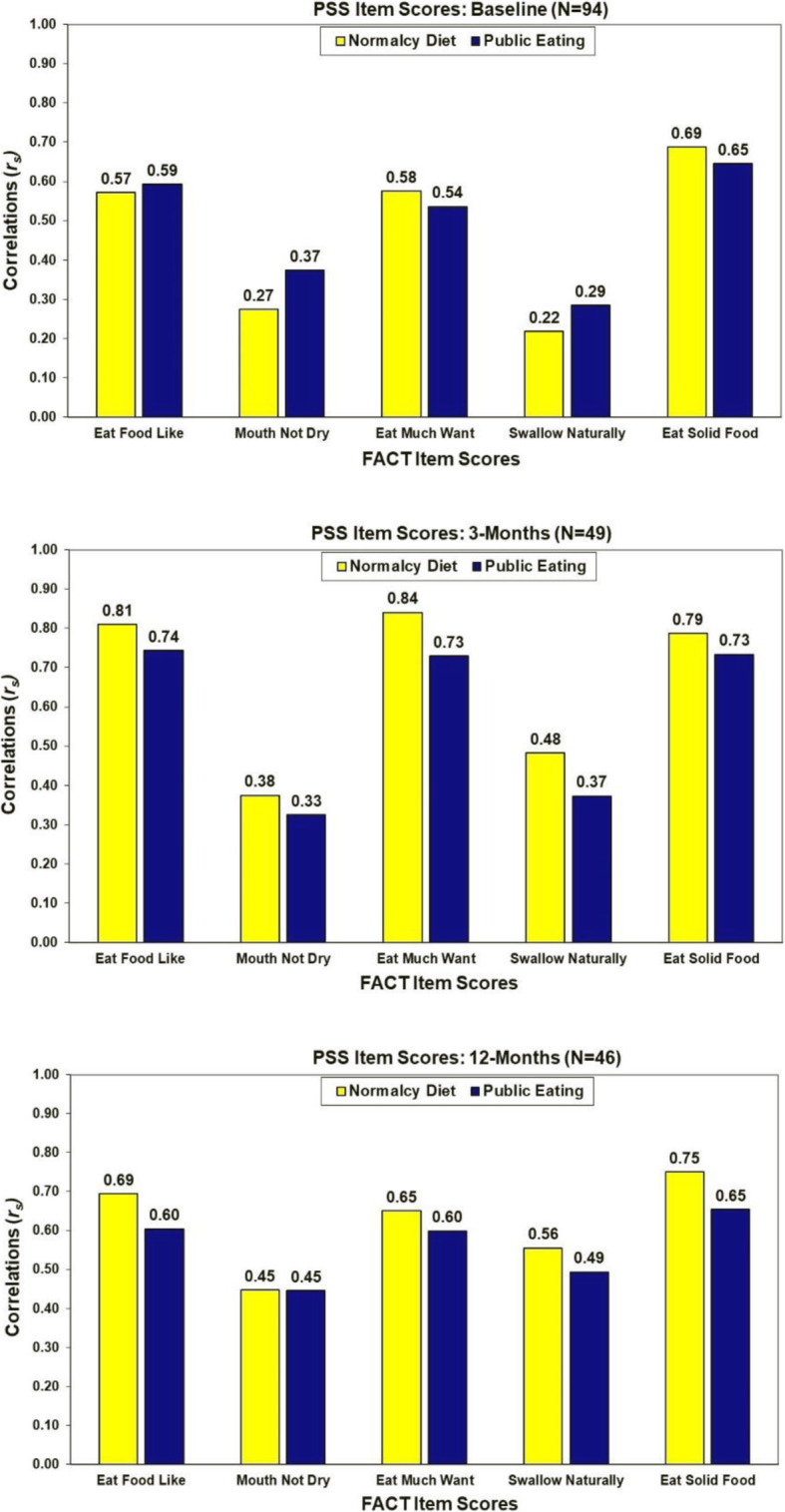
Correlations of FACT Swallow Items with PSS Swallow Items at Baseline

Correlations between the two PSS swallow items were 0.70 at baseline (*N* = 97, *p* < 0.001), 0.84 at 3 months (*N* = 61, *p* < 0.001) and 0.80 at 12-months (*N* = 55, *p* < 0.001). Associations of the FACT items tapping into swallowing function with the PSS item scores are summarized in Fig. 2. Consistently the strongest correlations were observed between the PSS items and the self-reported FACT items of “eating food I like” (*r*_*s*_ = 0.57 to 0.81), “eat as much as I like” (*r*_*s*_ = 0.54 to 0.84), and “eat solid foods” (*r*_*s*_ = 0.65 to 0.79).

#### Correlation of QOL with measures of swallow function

**Fig. 3:**
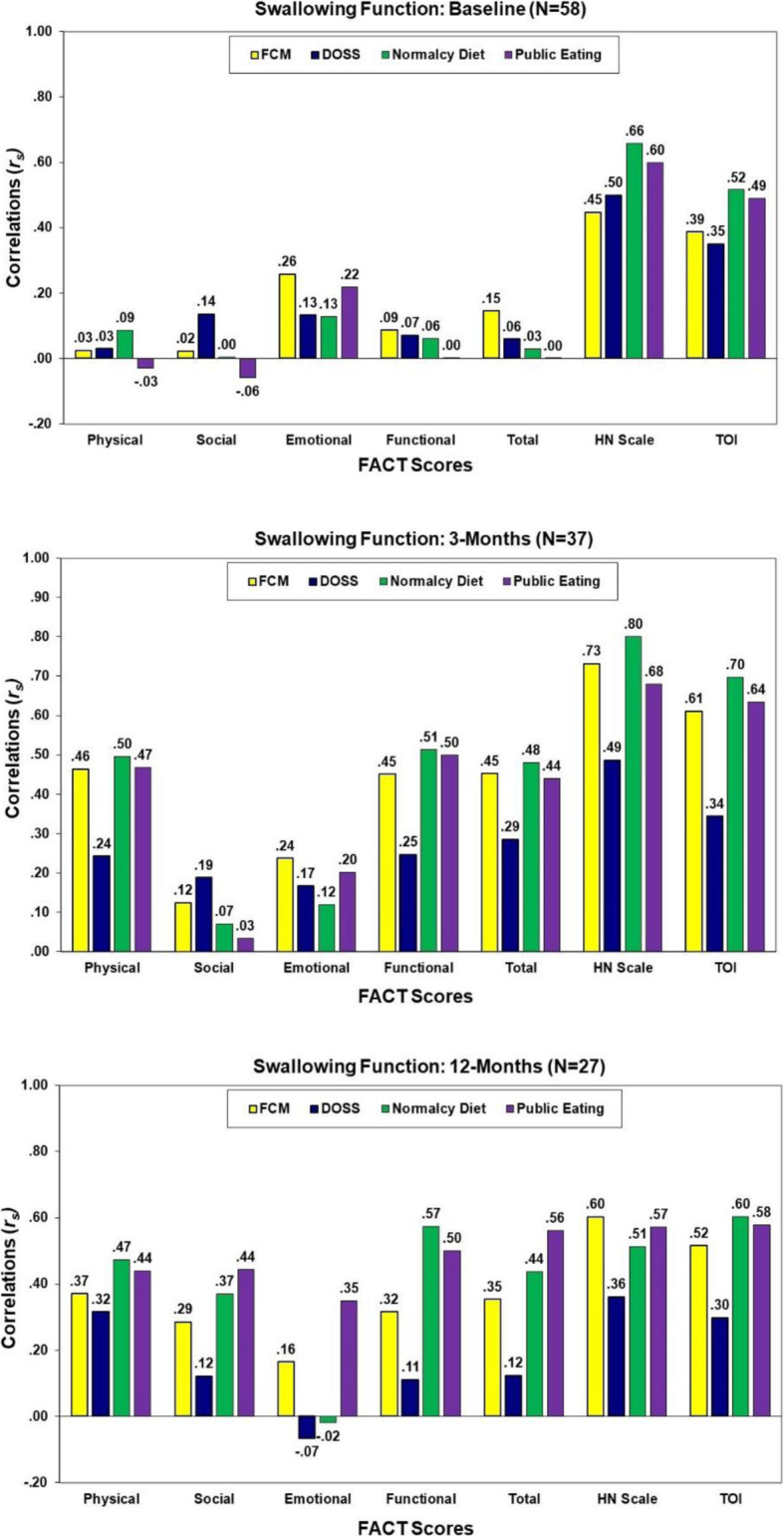
Correlations of the FACT scores with swallowing assessments at baseline, 3, and 12 months post-treatment

As shown in Fig. 3, with the exception of the FACT-HN and TOI, very low correlations of the FACT subscale scores with the swallow functioning scores were observed at baseline (*r*_*s*_ = − 0.06 to 0.26, *p* > .05). In general, the strongest associations of QOL with swallowing function at 3- and 12-months post-treatment were QOL as assessed by the FACT-HN and TOI (*r*_*s*_ = 0.30 to 0.80). Some increasing associations of the FACT overall and subscale scores with PSS scores were observed at 12-months.

#### Associations of objective swallow function with survival

Summaries of the scores on the FCM and DOSS at entry into the study and at 3-months post-treatment for patients who survived to 12 months and those who did not are presented in Table 6. As expected, lower FCM and DOSS scores at baseline were associated with increased likelihood of death during the study period. Approximately 30% of those deceased had FCM and DOSS values in the ‘Severe’ to ‘Moderate’ (i.e., <= 4) at baseline compared to only 6% (DOSS) and 3% (FCM) of other patients (*p* < =0.003). The associations with the 3-month assessments were mixed with a statistically significantly association of the DOSS scores with survival (*p* = 0.001) but not with the FCM (*p* = 0.147) (see Table 6).

**Table 6:** Baseline and 3-months swallowing function scores of the patients who survived and those who did not

Swallow Measure	Total N (%)	Alive N (%)	Deceased N (%)	*p*-value
DOSS (Baseline)	*N* = 92	*N* = 70	*N* = 22	0.003
Severe	4 (4.3)	0 (0.0)	4 (18.2)	
Moderate	7 (7.6)	4 (5.7)	3 (13.6)	
Mild	62 (67.4)	50 (71.4)	12 (54.5)	
Normal	19 (20.7)	16 (22.9)	3 (13.6)	
FCM (Baseline)	*N* = 94	*N* = 71	*N* = 23	< 0.001
Severe	4 (4.3)	0 (0.0)	4 (17.4)	
Moderate	5 (5.3)	2 (2.8)	3 (13.0)	
Mild	16 (17.0)	10 (14.1)	6 (26.1)	
Normal	69 (73.4)	59 (83.1)	10 (43.5)	
DOSS (3 Months)	*N* = 69	*N* = 53	*N* = 16	0.001
Severe	12 (17.4)	5 (9.4)	7 (43.8)	
Moderate	14 (20.3)	9 (17.0)	5 (31.2)	
Mild	43 (62.3)	39 (73.6)	4 (25.0)	
Normal	0 (0.0)	0 (0.0)	0 (0.0)	
FCM (3 Months)	*N* = 39	*N* = 37	*N* = 2	0.392
Severe	2 (5.1)	2 (5.4)	0 (0.0)	
Moderate	3 (7.7)	2 (5.4)	1 (50.0)	
Mild	33 (84.6)	32 (86.5)	1 (50.0)	
Normal	1 (2.6)	1 (2.7)	0 (0.0)	

### Voice

The objective of the voice assessments were to conduct a longitudinal assessment of voice function in patients undergoing concurrent chemoradiation. The patient self-reported Voice Handicap Index (VHI) and physician-scored Voice Desirability Assessment (VDA) scores summarized in Table 7 illustrate a different pattern for patients with larynx compared to oropharynx. There appeared to be a general decrease VHI scores from baseline to 12-months albeit the pattern was not statistically significant (*p* = .124). On the other hand, those with oropharynx cancer demonstrated a statistically significant pattern (*p* = .003). VHI scores improved from baseline to 3-months (post-hoc *p* = .001) yet decreased by 12-months to the point that the improvement was no longer statistically significantly different from baseline (post-hoc *p* > .05). While the pattern was one of improvement in VDA scores from baseline at both 3-months and 12-months for patients with larynx cancer, there were only 8 patients available for that analysis and the findings were not statistically significant (*p* = .239). In the 29 oropharynx patients available, there was a statistically significant difference among the VDA assessments (*p* = .010). A decrease in VDA scores from baseline was apparent at 3-months and remained at 12-months (post-hoc *p* = .008 and .007 respectively) (Table 7).

**Table 7:** Voice longitudinal summaries over time

Voice Measure	Baseline Median (IQR)	3-Months Median (IQR)	12-Months Median (IQR)	*p*-value
VHI				
Overall (*n* = 43)	6.0 (1, 24)	18.0 (6, 33)	11.0 (0, 32)	.124
Larynx (*n* = 8)	45.5 (4, 51)	15.0 (0, 41)	20.0 (0, 63)	.061
Oropharynx (*n* = 35)	5.0^a^ [1, 14]	18.0^b^ (6, 32)	10.0 (1, 24)	.003
VDA				
Overall (*n* = 37)	45.0 (37, 50)	40.0 (30, 47)	45.0 (32, 49)	.147
Larynx (*n* = 8)	20.0 (4, 43)	40.0 (30, 45)	38.5 (25,48)	.239
Oropharynx (*n* = 29)	47.0^a^ (40, 50)	40.0^b^ (30, 49)	45.0^b^ (35, 50)	.010

Note: Superscripts indicate statistically significant Bonferroni-corrected pairwise differences in post-hoc analyses

## Discussion

The results of this study confirm previous reports indicating that QOL declines as a result of CCR; however, by twelve months post-treatment, QOL returned towards normal. QOL did not differ among those proceeding to CCR versus those who required salvage surgery, although this is probably related to the relatively small number of individuals receiving surgery (*n* = 13) compared to CCR (*n* = 95). Our results add to a growing literature that demonstrates the ability of QOL scores to predict overall survival.

Using both patient report and objective measures of swallow function, patients treated on E2399 demonstrated a marked decline in swallow function 3 months after completion of chemoradiation. Swallow function returned toward baseline by 12 months post treatment. Less than 10% of patients had severe dysphagia at 12 months as scored using either the FCM or the DOSS, and very few patients were gastrostomy-tube dependent at 12 months. This compares favorably to the incidence of 12 month gastrostomy-tube dependence reported in other CCR studies (NRG RTOG 0522 17.2–21.2%; NRG RTOG 0129 18.9–26.1%). The improvement in post-treatment swallow function may be attributed to several factors. First, this group of patients had relatively early stage disease; by definition, patients were deemed resectable at the time of study entry. Second, patients were seen by SLP periodically throughout the course of their therapy. This allowed the SLPs to have early and constant contact with patients, potentially improving overall outcomes. Third, the concurrent regimen used in this study was mild and may have resulted in less late-effect fibrosis with associated pharyngeal dysfunction.

The DOSS scores were consistently lower than the FCM scores, an expected finding given that DOSS measures function irrespective of the need for compensatory mechanisms, while the FCM measures actual disability. The FCM demonstrated consistently higher correlations with both the PSS and FACT-HN swallow items compared to the DOSS. The correlations between the FCM and self-report swallow items on the PSS and FACT-HN appear to be sufficiently strong to justify their use as a surrogate marker for swallowing function in large therapeutic trials.

Decreased swallowing function post treatment was also associated with increased mortality. It is unclear whether this is a causal relationship. It may be reasonably hypothesized, however, that dysphagia and its complications (such as aspiration) may directly impact on survival.

Our paper has several limitations. Firstly, as a trial that was initiated in a cooperative group setting in 1999, the majority of centers did not employ intensity-modulated radiation therapy (IMRT), and radiation doses to salivary glands and pharyngeal constrictors were higher than it would be in contemporary practice. However, a recent examination of two-year dysphagia rates and related outcomes in the Surveillance, Epidemiology, and End Results-Medicare database for 2002–2011 demonstrated that rates of dysphagia (45.3%), stricture (10.2%), and pneumonia (26.3%) remain high, and that indeed the rate of dysphagia had increased in the IMRT era [17]. Second, this was a small study including two distinct subsites and including both patients with HPV-associated and non-HPV-associated oropharynx cancer, with their differing age of onset, co-morbidity burdens, and natural history. Finally, we collected patient-reported and functional data for only 2 years following completion of CCR. It is now recognized that a subset of head and neck cancer patients develop late dysfunction, which may be progressive even after 7 years from the completion of radiation [18, 19].

## Conclusions

Swallow function and voice quality decline as a result of high dose radiation, and is exacerbated by radiosensitizing chemotherapy. Favorable functional results were obtained with this cisplatin-sparing function preservation strategy in a study which was designed in 1999 and in which the majority of patients did not receive radiation with intensity-modulated approaches. The contemporary use of IMRT may result in even superior functional outcomes due to the ability to spare salivary tissue and pharyngeal constrictors, and contemporary treatment deintensification trials in oropharynx cancer such as NRG Oncology ROTG 1016 have included careful functional assessment as well [18].

The FACT-HN swallow questions and the PSS-HN swallow questions demonstrate a decrease in swallowing function three months post treatment with a return towards normal 12 months post-treatment, and that severe swallowing abnormalities were found in less than 10% of patients at 12 and 24 months. The FCM correlated well with the FACT-HN and the PSS-HN swallow items*.* The correlation between these two self-report measures is high, and data are sufficiently granular to justify the use of these questions to measure swallowing disability in future trials.

Voice assessment instruments VHI and VDA shows a strong correlation, indicating either patient or physician perception to be reasonable options in future head and neck studies.

These findings suggest that PROs have practical utility in assessing swallowing function outcome and may be used as a surrogate for objective measures of swallow function. Ultimately the goal is to utilize future data from these instruments prospectively to predict which patients are most likely to develop swallowing and speech difficulties, and to intervene with appropriate therapy.

## Data Availability

The datasets used and/or analyzed during the current study are available from the corresponding author on reasonable request.

## Abbreviations

CCR: Concurrent chemoradiation
QOL: Quality of life
FACT-HN: The Functional Assessment of Cancer-HN
PSS-HN: Performance Status Scale – Head and Neck
PEG: percutaneous gastrostomy tube.
EORTC: European Organization for Research and Treatment of Cancer
OP: Oropharynx
ECOG-ACRIN: Eastern Cooperative Oncology Group-American College of Radiology Imaging Network
DOSS: Dysphagia Outcome Severity Scale
FCM: Functional Communication Measure
RTOG: Radiation Therapy Oncology Group
VDA: Voice Desirability Assessment
SLP: Speech and language pathologist
PRO: Patient reported outcome
